# Nanoporous 3D-Printed Scaffolds for Local Doxorubicin Delivery in Bone Metastases Secondary to Prostate Cancer

**DOI:** 10.3390/ma11091485

**Published:** 2018-08-21

**Authors:** Pouyan Ahangar, Elie Akoury, Ana Sofia Ramirez Garcia Luna, Antone Nour, Michael H. Weber, Derek H. Rosenzweig

**Affiliations:** 1 Division of Orthopedic Surgery, McGill University, Montreal, QC H3G 1A4, Canada; pouyan.ahangar@mail.mcgill.ca (P.A.); elie.akoury@mail.mcgill.ca (E.A.); A.Ramirez@stud.uni-heidelberg.de (A.S.R.G.L.); antone.nour@mail.mcgill.ca (A.N.); michael.weber@hotmail.com (M.H.W.); 2 Medical Faculty Mannheim, Heidelberg University, D-68167 Heidelberg, Germany; 3 The Research Institute of the McGill University Health Centre, Montreal, QC H3H 2L9, Canada; 4 Montreal General Hospital C10.148.6, 1650 Cedar Ave, Montreal, QC H3G 1A4, Canada

**Keywords:** low-cost 3D printing, nanoporous filament, bone metastases, doxorubicin, local delivery, prostate cancer, bone substitute

## Abstract

The spine is the most common site of bone metastasis, often originating from prostate, lung, and breast cancers. High systemic doses of chemotherapeutics such as doxorubicin (DOX), cisplatin, or paclitaxel often have severe side effects. Surgical removal of spine metastases also leaves large defects which cannot spontaneously heal and require bone grafting. To circumvent these issues, we designed an approach for local chemotherapeutic delivery within 3D-printed scaffolds which could also potentially serve as a bone substitute. Direct treatment of prostate cancer cell line LAPC4 and patient derived spine metastases cells with 0.01 µM DOX significantly reduced metabolic activity, proliferation, migration, and spheroid growth. We then assessed uptake and release of DOX in a series of porous 3D-printed scaffolds on LAPC4 cells as well as patient-derived spine metastases cells. Over seven days, 60–75% of DOX loaded onto scaffolds could be released, which significantly reduced metabolic activity and proliferation of both LAPC4 and patient derived cells, while unloaded scaffolds had no effect. Porous 3D-printed scaffolds may provide a novel and inexpensive approach to locally deliver chemotherapeutics in a patient-specific manner at tumor resection sites. With a composite design to enhance strength and promote sustained drug release, the scaffolds could reduce systemic negative effects, enhance bone repair, and improve patient outcomes.

## 1. Introduction

Bone metastases, especially to the spine [1], are the most common type of bone cancer [2]. These are tumors whose origin is outside the spine and include: breast [3], prostate [4], lung [5], gastrointestinal, thyroid [6], as well as tumors associated with the hematopoietic system such as lymphoma and myeloma. With advancements in medical, radiation, and surgical oncology, these patients are living longer, and the disease burden is growing [7,8]. While radiotherapy can be applied, surgical excision is the primary treatment for bone tumors in the spine with instability and or neurological deficits, and is often extensive to ensure removal of all malignant tissue and prevent tumor recurrence [9]. The best technique for surgically treating a spine metastasis has yet to be determined [2], and the resulting defect is often large and will not heal spontaneously. Postero-lateral fusion with autologous bone graft is preferred for fusion but may not address the structural defect. Furthermore, harvest of autograft is associated with well recognized limitations including donor-site morbidity and increased surgical and recovery time [10]. The quantity of autograft bone from various sites is usually insufficient to fill the large defects generated from tumor removal. Therefore, bone substitutes are an attractive alternate for such cases.

When tumors are more advanced or unresectable, systemic delivery is of chemotherapeutics such as paclitaxel/docetaxel, cisplatin, and doxorubicin is a major strategy for treating oligometastatic bone cancer [11]. These agents are typically administered in combination with surgery as an adjuvant for local control at the surgical site or the treatment of other sites. While chemotherapeutics can effectively limit proliferation of tumor cells, high systemic doses are required for their appropriate activity at the tumor sites. The high doses are associated with negative side effects such as cardiac toxicity (doxorubicin) [12], kidney toxicity (cisplatin) [13], and neurotoxicity (paclitaxel) [14]. These therapeutics can also affect bloods counts, impacting the immune system and rendering patients more susceptible to infections. Therefore, local application of therapeutics is an attractive alternate to delivering high local doses without the side effects of systemic delivery. We have previously reported on benefits of local bisphosphonate delivery in preventing tumor induced osteolysis [15]. Furthermore, tumor recurrence at the surgical site with or without nonunion and hardware failure is a significant complication status post-tumor resection and reconstruction which might be augmented by local delivery of adjuvant chemotherapy. Major efforts have been made to develop local chemotherapeutic delivery methods to control the release and concentration of drug at the tumor site. Polymers, hydrogels, liposomes, and nanoparticles are some recent examples of such delivery vehicles [16], which if successfully commercialized, hold an advantage over systemic chemotherapy and could potentially address an unmet clinically need.

Among the different approaches for local drug delivery, 3D printing (or additive manufacturing) holds a high level of intrigue. The approach is to use 3D printed scaffolds to precisely promote bone repair and provide therapeutic delivery to block cancer recurrence [17]. First described in the mid-1980s by Charles Hull [18], this layer-by-layer manufacturing method constructs complex solid objects from computer-generated models. It is particularly interesting for applications such as orthopedic surgery, where damaged tissue or defects can be replaced with anatomically matched, patient-specific implants. Indeed, the pharmaceutical industry and academics alike have shown enthusiasm for using additive manufacturing for drug delivery devices [18]. Over the past 5 years, the cost of desk-top and industrial 3D printers has significantly decreased allowing rapid increases in research efforts. We have previously shown feasibility of low-cost 3D-printing in orthopedic tissue engineering applications [19], and other groups have shown feasibility of 3D printing for bone substitutes [20,21,22]. Several new types of materials have become commercially available for use in low-cost 3D printers, including flexible, dissolvable, and porous polymers. The PORO-LAY series of 3D print filaments is a thermoplastic polyurethane (TPU) copolymer with polyvinyl alcohol (PVA). After printing, the PVA component can be dissolved in a water bath leaving a nanoporous and sponge-like TPU network which has been shown to be useful for biofuel cell development [23], particle filtration [24], and modeling elastic tissues [25]. The PORO-LAY filaments are available as LAY FOMM 60, LAY FOMM 40 and GEL LAY displaying increasing porosity, respectively. It is important to note that for clinical applications, both structural and nonstructural grafts with instrumentation can be used. The PORO-LAY series of materials would then be considered nonstructural. In this study we set out to harness the sponge-like properties of 3D printed PORO-LAY constructs for local delivery of chemotherapeutics.

The PORO-LAY filaments are mechanically inferior to standard bone substitutes. However, they possess high potential for bioactive substance uptake and release, can be printed into complex geometries and can be compositely printed with mechanically competent polymers. Therefore, exploration of chemotherapeutic delivery with these materials is a necessary initial step toward future development into bone substitutes which also limit cancer recurrence. Here, we hypothesized that these materials could be readily loaded with doxorubicin (DOX) and that the GEL LAY (having the highest porosity) would release the most DOX. DOX was chosen as a model drug due to its widespread use in bone cancer. The EC50 for DOX treatment of prostate cancer cell line LAPC4 and patient-derived spine metastases cells was determined. DOX-loaded scaffolds were applied to LAPC4 and patient-derived metastases cells with all scaffold types able to inhibit cell metabolic activity and proliferation. We show feasibility of using low-cost 3D printed nanoporous scaffolds for DOX delivery, which could potentially be applied directly at tumor resection sites to fill the bone defects while limiting cancer recurrence.

## 2. Materials and Methods

### 2.1. Cell Lines and Patient-Derived Cells

The prostate cancer cell line LAPC4 was provided by Dr. Jacques Lapointe’s Laboratory, at the Research Institute of McGill University Health Centre (RI-MUHC). Collection of patient samples was approved by the institutional review board of McGill University (IRB# A08-M78-13B titled The Epidemiology, Process, and Outcomes of Spine Oncology). Resected metastatic spine tumors secondary to prostate cancer were collected with consent from a patient undergoing surgery at the Montreal General Hospital. Tissue samples were processed by first washing in phosphate buffered saline (Sigma, Burlington, ON, Canada) and then cut into 5 mm × 5 mm sections and incubated at 37 °C overnight in a collagenase type II (Thermofisher, Gibco, Burlington, ON, Canada). Digested cells were strained in a 100 µM cell strainer, and then pelleted in a centrifuge at 300× *g* for 5 min. Isolated cells consisting of a mix population of bone metastasis-derived patient cells and bone cells were cultured in RPMI cell culture medium (Gibco, Thermofisher) supplemented with 10% fetal bovine serum (Gibco, Thermofisher), 1% antibiotics (Gibco, Thermofisher), 1% glutamax (Gibco, Thermofisher), and 1% fungizone (Gibco, Thermofisher) at 37 °C in a humidified atmosphere of 5% CO_2_.

### 2.2. Metabolic Activity and Proliferation Assays

Cell metabolic activity and proliferation were assessed using a commercial Alamar Blue^®^ kit and a Vybrant^®^ MTT kit both according to the manufacturer’s instructions (both from Thermofisher). Briefly, LAPC4 and patient derived cells were seeded in Costar 96 well plates (FisherScientific, Burlington, ON, Canada) coated with 0.1% poly-l-lysine (Sigma) at a density of 5000 cells/well and were grown in standard conditions (RPMI, 10% FBS, 1% penicillin/streptomycin) for 24 h. The next day, cells were treated with either sterile PBS vehicle or doxorubicin (Sigma) in low-serum conditions (1% FBS) for 7 days. The media was replaced on day 4 for each experiment. For Alamar Blue assay, Almar Blue dye was added to media at 1:10 dilution on day 7, and cells were incubated at 37 °C for 4 h. For the MTT assay, the cells were labelled with MTT at 1:10 dilution on day 7 and incubated for 4 h at 37 °C. Then, 75 µL of media containing MTT was removed from each well before adding 50 µL of DMSO (Sigma) for each well and incubating for 10 min at 37 °C. After incubation, fluorescence of Alamar Blue (Excitation–540 nm, Emission 585) or the absorbance of MTT (540 nm) was analyzed using the Tecan Infinite M200 pro microplate reader (Tecan Trading, AG, Männedorf, Switzerland). For experiments testing drug-loaded scaffolds, 24-well plates (Sarstedt, Montreal, QC, Canada) were seeded with 20,000 LAPC4 or patient derived cells/well. After 24 h of equilibration in standard culture media, fresh media was replaced and the PORO-LAY scaffolds containing either 50 ng of doxorubicin or PBS vehicle were added to the wells in triplicate. Three independent experiments were performed for each cell type.

### 2.3. Live/Dead Viability Assay

Live/dead assay (ThermoFisher, Burlington ON, Canada) was performed according to manufacturer’s instructions, and as previously reported [26,27]. Images were captured using an Olympus IX73 inverted fluorescence microscope. Live and dead cells were quantified using Image J software (NIH, v.1.6.0, Bethesda, MD, USA).

### 2.4. Boyden Chamber Migration Assay

To test migration, LAPC4 cells were seeded at a density of 20,000 cells/well in the upper compartment of Boyden trans-well chambers with polycarbonate filter inserts (8 µm pore size; Corning, NY, USA) coated with poly-l-lysine. After 24 h, cells were treated with PBS vehicle or doxorubicin at different concentrations in low-serum conditions (1% FBS) in the upper compartment. Cell migration was triggered over 7 days using RPMI supplemented with 2% FBS media as a chemoattractant in the lower compartment. After migration through the filters, the cells of both compartments were assayed for Alamar Blue^®^ to check for metabolic activity. The cells of the upper compartment of the insert were then removed with cotton swabs, and those on the lower compartment were fixed with 4% paraformaldehyde (Thermofisher, Burlington, ON, Canada), counterstained with DAPI (Sigma), and imaged using an inverted microscope (Olympus, IX71, Richmond Hill, ON, Canada) before counting. Each treatment was done in triplicate, with quantification averaged from five random fields for each sample.

### 2.5. 3D Printing of Scaffolds

**Figure 1:**
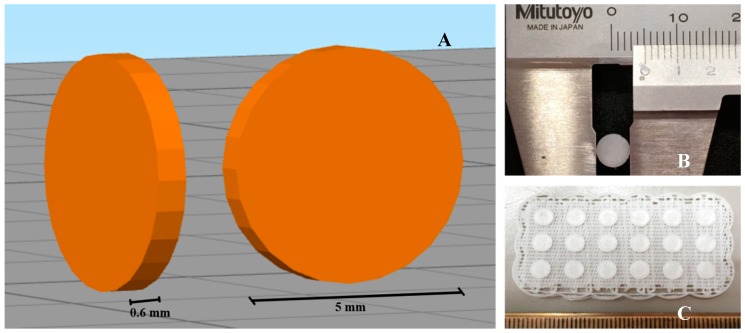
3D printed polymer disc. (**A**) 3D rendering of a polymer disk. (**B**) Close-up of a printed disc. (**C**) Disks are printed in sets of 18 on a scaffold. They are carefully removed from the scaffold for further processing.

3D printing of scaffolds was carried out as previously described [19]. Briefly, 3D models of 0.6 mm height and 5 mm diameter were generated in SketchUp software (Trimble, Brossard, QC, Canada), and models were sliced into G-code using Simplify 3D software (Simplify 3D, Cincinnati, OH, USA) (Figure 1). PORO-LAY series of filaments (1.75 mm) Lay FOMM 60, 40 and Gel Lay were purchased from Matterhackers (Burbank, CA, USA). All prints were made using a Flashforge Creator Pro (Flashforge Corp., Zhejiang, China), with a 0.3 mm nozzle, and print settings of 220 °C print temperature, 50 °C bed temperature, and 18 mm/sec speed. All scaffolds from the 3 different materials were washed in 6 changes of distilled water over 3 days prior to use, and then washed in 70% ethanol for 5 min and exposed to UV light for 15 min/side for disinfection. Doxorubicin was resuspended in sterile PBS at 10 mg/mL stock concentration. Doxorubicin loading on scaffolds was performed in a laminar flow safety cabinet, in 5 µL applications (with desired concentration), which typically dried within 5–10 min.

### 2.6. Assessment of Doxorubicin Release from Scaffolds

Doxorubicin was diluted to 100 ng/µL, and 500 ng was loaded onto the Lay FOMM 60, 40, and Gel Lay scaffolds. Individual scaffolds were placed in 1.5 mL microfuge tubes, and 200 µL of PBS was added. At 4 h, 8 h, 24 h, and then daily for 7 days, 100 µL was removed and stored, and 100 µL fresh PBS was replaced. Doxorubicin standard curves were prepared. Standards and collected samples were loaded into a Corning 96-well black microplate with clear bottom (Sigma) and fluorescence was measured using fluorescence detection modality with illumination at 480 nm and peak emission at 570 nm using a Tecan Infinite Pro M200 microplate reader (Tecan Inc., Morrisville, NC, USA). Quantification of amounts of doxorubicin released from 3 independent experiments was determined by extrapolation from standard curves using GraphPad Prism 5.0 software (GraphPad, La Jolla, CA, USA). For experiments where scaffolds are loaded with DOX for cell treatment, 50 ng of DOX in 5 µL of PBS was applied to each scaffold under sterile conditions. These DOX loaded samples were then placed individual wells of 24-well plates seeded with either 50,000 LAPC4 or patient derived cells. The scaffolds slightly float, and never touched the surface of the dish. After 7 days of treatment, metabolic activity and proliferation assays were performed, as described above.

### 2.7. Scanning Electron Microscopy

Field emission scanning electron microscopy of the 3D printed scaffold surfaces before and after washing was performed using a JEOL JSM-7400F (JEOL Ltd., Tokyo, Japan) operating at 2 kV. High resolution images were taken to visualize the nano-porosities of the different materials.

### 2.8. Statistical Analysis

Statistical analyses were performed using Microsoft Excel 2016 (Version 1803, Build 9126.2259) through a one-tailed non-paired Student’s *t*-test between each individual variable and its control. All data are expressed as the mean ± SD. Comparisons were made by ANOVA (with or without White’s adjust depending on heteroscedasticity) and Tukey post-hoc test at a 95% confidence level. P-values lower than 0.05 were considered as statistically significant.

## 3. Results

### 3.1. Evaluation of Effective Doxorubicin Dosage for LAPC4 Cells

**Figure 2:**
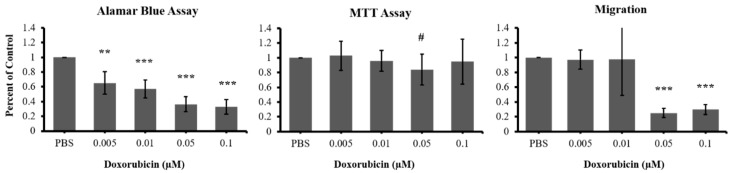
Effect of DOX on LAPC4 metabolic activity and migration. Metabolic activity was significantly decreased with all the drug concentrations tested. There was no effect on LAPC4 proliferation with DOX treatment. Migration ability of LAPC4 cells was significantly decreased with only the higher concentrations of DOX (0.05 and 0.1 µM). Alamar Blue and MTT assays were done as triplicates per trial over four independent experiments. The migration assay was done as triplicates per trial in three independent experiments. Results are shown as mean ± STDEV (** = *p* < 0.01, *** = *p* < 0.001, ^#^ denotes *p* = 0.08).

**Figure 3:**
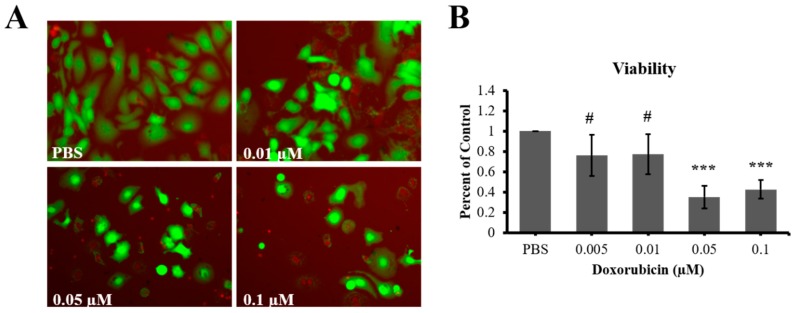
LIVE/DEAD cell viability analysis of LAPC4 cells. (**A**) DOX treatment decreases the amount of live (green) cells. (**B**) DOX treatment significantly decreases viability with higher doses compared to PBS control. LIVE/DEAD assay was done in three independent experiments each in triplicate. Results are shown as mean ± STDEV (*** = *p* < 0.001, ^#^ denotes *p* = 0.06).

To date, the effect of doxorubicin on the prostate cancer cell line LAPC4 has not been studied. We therefore tested the effect of direct DOX treatment on the metabolic activity of LAPC4 using a range of low concentrations (0.005 µm–0.1 µm) over 7 days (Figure 2). Because different types of metabolic activity assays have different sensitivities when treating with cytotoxic drugs [28], we applied two metabolic activity assays: the more sensitive Alamar blue assay and the less sensitive MTT assay. Following treatment, we observed a statistically significant, dose dependent decrease in metabolic activity of LAPC4 cells (*p* < 0.01). The EC_50_ of DOX for LAPC4 metabolic activity was determined to be 0.01 µm, as Alamar blue assay showed a reduction in metabolism of roughly half (43% ± 12%, *p* < 0.001) compared to control. To determine whether the reduced metabolic activity was due to a decreased proliferation, DOX treated LAPC4 cells were assessed by MTT assay which tests redox enzyme activity which correlates with proliferation rate of cells. Surprisingly, we observed that LAPC4 cells did not show a significant difference in the proliferation rate following DOX treatment when compared to control (Figure 2). The EC_50_ data (0.01 µM DOX) is summarized in Table 1, suggesting different sensitivities of Alamar and MTT assays for this cell type. To investigate whether the decrease in metabolic activity was due to a decrease in the total cell number, we conducted a LIVE/DEAD cell viability assay on LAPC4 cells following direct treatment with the same range of DOX concentrations. We observed a dose-dependent reduced number of live cells in response to DOX treatment (Figure 3). The LIVE/DEAD assay also revealed a reduction in total cell counts for DOX treated cells versus control. Taken together, the data suggests DOX slows proliferation of the cells without being overwhelmingly toxic, leading to a decrease in total cell number, and ultimately a reduction in metabolic activity of LAPC4. Furthermore, these data indicate that Alamar assay may be more appropriate when determining the effects of lower doses of chemotherapeutics.

We also assessed the migration ability of LAPC4 following DOX treatment in the same conditions described above using the Boyden chamber assay. We observed that the migration of LAPC4 cells across a cell-permeable membrane was significantly decreased with the 2 highest doses (0.05 µm and 0.1 µm) by more than 70% (±7%, *p* < 0.001) compared to control (Figure 2). We did not observe a significant decrease in lower doses, specifically in the EC_50_ dose of 0.01 µm established earlier for metabolic activity.

### 3.2. 3D Scaffold Structure

**Figure 4:**
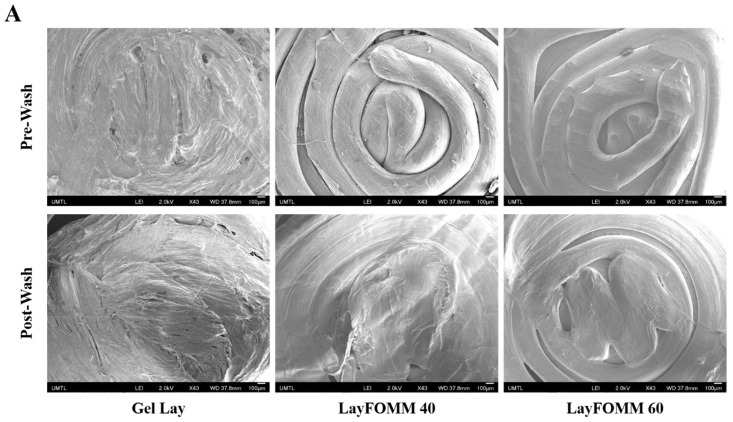

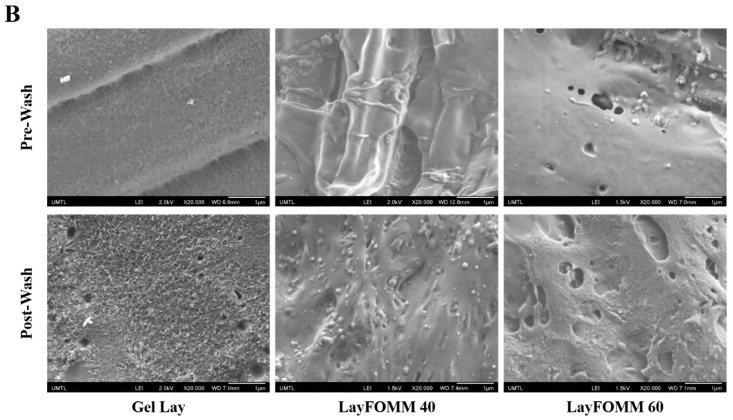
Electron microscope images of polymers. (**A**) 43× magnification showing polymer surface texture before and after wash with DI water with a clear change in surface texture after wash. (**B**) 20,000× magnification showing polymer surface texture before and after wash with DI water with increased porosity noted in Gel Lay after wash.

After establishing the effect of direct treatment of DOX on the metabolic activity and migration ability of LAPC4, we sought to describe the surface structure and porosity of the Lay FOMM 60, 40, and Gel Lay scaffold materials directly before and after washing out the polyvinyl alcohol component which will be impregnated with Dox and eventually tested for drug release on LAPC4 cells. At a 43× magnification, SEM images show some clear differences in the Gel Lay scaffolds after 3 days of washing in DI water (Figure 4A). The PVA component of the polymer increases from LAY FOMM 60 to Gel LAY (right to left) (Figure 4A). Higher magnification (20,000×) shows increased porosity in all washed scaffolds compared to their pre-washed counterparts. Furthermore, Gel Lay clearly shows the most surface porosity (Figure 4B).

### 3.3. 3D Printed Scaffold DOX Release

**Figure 5:**
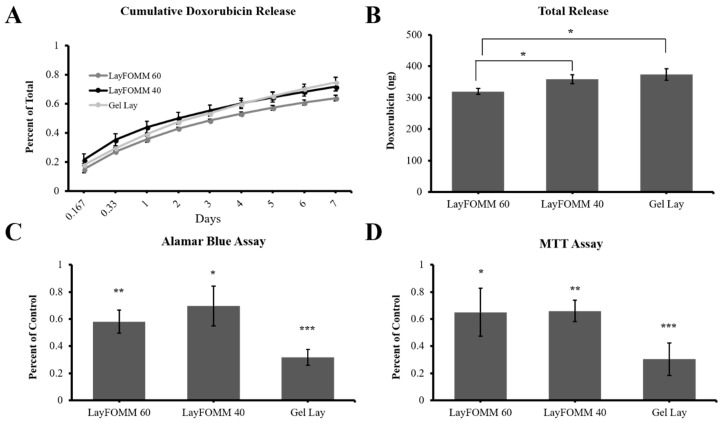
3D printed scaffolds for DOX release. (**A**) 3D printed polymer discs loaded with 500 ng of DOX can sustainably release more than 50% of loaded drug into solution by day 7. (**B**) Total amount of Dox released by each polymer with LayFOMM 40 and Gel Lay releasing more than LayFOMM 60. (**C**) Alamar assay for metabolic activity of LAPC4 cells treated with 50 ng DOX-loaded scaffolds for 7 days. All three polymers released levels of DOX adequate for inhibiting metabolic activity, with Gel Lay releasing the most. (**D**) MTT assay for metabolic activity of LAPC4 cells treated with DOX loaded polymers for 7 days with Gel Lay releasing the most amount of drug. Three independent experiments were performed, with error bars representing standard deviation between each experiment (* = *p* < 0.05, ** = *p* < 0.01, *** = *p* < 0.001).

Upon washing the Lay FOMM 60, 40, and Gel Lay materials with water, the polymer transforms to a soft sponge-like material with the ability to absorb and release aqueous solutions. We took advantage of this property and infused an aqueous DOX solution (loading a total amount 500 ng) into the polymer and assessed its ability to release the drug into PBS over 7 days (Figure 5A). All 3 scaffolds were able to release more than 50% of the initially loaded drug amount. The Gel Lay material significantly releases the highest amount as expected due to its increased porosity (Figure 5A,B). Both Lay FOMM 40 and Gel Lay released a statistically significant larger amount of drug compared to LAY FOMM 60. However, there was no difference between the drug amount release between the Lay FOMM 40 and Gel Lay polymers. This indicates that drug release can be tuned by using the different polymers or combinations of the polymers by using multihead 3D printers.

### 3.4. 3D Scaffold Drug Delivery to LAPC4 Cells

Having established the DOX release kinetics by these polymers, we conducted an experiment to assess the effectiveness of the polymers in delivering drug to cultured LAPC4 cells. Based on the kinetics profiles, we determined that loading the scaffolds with 50 ng of DOX would be sufficient to achieve a concentration of 0.01–0.025 µM in 5–7 days of treatment (depending on the scaffold type). LAPC4 cells were treated with 50 ng DOX-loaded polymers (Lay FOMM 40, 60, and Gel Lay) over 7 days before assaying for metabolic activity and proliferation compared to cells given scaffolds loaded with PBS vehicle. Alamar blue assay showed that metabolic activity of LAPC4 was significantly decreased with all 3 types of DOX loaded materials. Lay FOMM 60 caused a 42 ± 8% decrease (*p* = 0.001), Lay FOMM 40 caused a 31 ± 15% decrease (*p* = 0.024), and Gel Lay caused a 68 ± 6% (*p* = 0.0004) decrease in metabolic activity (Figure 5C). Similarly, MTT assay showed significantly reduced metabolic activity following treatment with all 3D scaffold types (Metabolic activity % decrease-Lay FOMM 60, 35 ± 18%, *p* = 0.026; Lay FOMM 40, 34 ± 8%, *p* = 0.002; Gel Lay, 70 ± 12%, *p* = 0.0006) (Figure 5D).

### 3.5. Direct Treatment and Scaffold Drug Delivery to Patient Derived Cancer Cells

**Figure 6:**
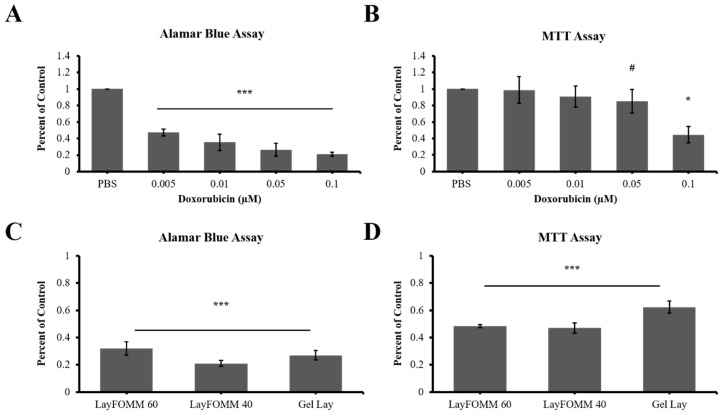
Patient derived spine metastases cells (secondary to prostate cancer) following direct and DOX-loaded scaffold treatment. (**A**) Direct treatment of patient derived cells with DOX showing significantly decreased metabolic activity with all the ranges tested over 7 days using Alamar assay. (**B**) MTT metabolic activity assessment of patient derived cells decreased with only the highest concentration of DOX. (**C**) 3D printed scaffold DOX release leading to decreased Alamar assessment of metabolic activity of patient derived cells over 7 days. (**D**) MTT assessment of metabolic activity of patient derived cells after treatment with 3D printed scaffold loaded with Dox for 7 days. Experiments were performed over a single trial in triplicate form cells isolated from a single donor. Error bars represent ± SD between each experiment (* = *p* < 0.05, *** = *p* < 0.001, ^#^ denotes *p* = 0.07).

After assessing the effectiveness of DOX-loaded materials on LAPC4, we sought to determine the effect of direct DOX treatment or DOX released from 3D printed scaffolds on patient derived cells. These cells were derived from resected prostate cancer spinal metastases tumors from a patient at our affiliated hospital. We first treated the patient derived cells with a range of DOX concentrations for 7 days. Interestingly, we observed the same statistically significant dose dependent response in metabolism as in LAPC4 following direct treatment. However, the patient derived cells were more sensitive than the LAPC4 EC_50_ dose of 0.01 µm, which reduced metabolic activity more than 50% (65 ± 10%, *p* = 0.00001) as shown by Alamar assay (Figure 6A). MTT assay however, showed that higher doses of 0.05 µm and 0.1 µm DOX treatment were required to reduce metabolic activity by 15 ± 14% (*p* = 0.07) and 56 ± 9% (*p* = 0.0003) of patient derived cells, respectively (Figure 6B).

Again, 50 ng of DOX was loaded to 3D printed scaffolds of the three materials, and then incubated with the patient derived cells over a 7-day period. Alamar assay showed that all three polymers significantly reduced cell metabolic activity by more than 65% (*p* < 0.0001 for all samples) compared to PBS loaded vehicle control scaffolds (Figure 6C). This decrease in metabolism was more prominent in patient derived cells in comparison to LAPC4 cells following DOX-loaded scaffold treatment, mostly due to the increased sensitivity of these patient derived cells. MTT assay also showed significantly reduced metabolic activity by more than 35% following DOX release from all three scaffolds (*p* < 0.001 for all 3) compared to controls (Figure 6D).

## 4. Discussion

The use of 3D printed scaffolds is an attractive approach to bone repair due to patient-specific customized geometries, use of resorbable polymers, low-cost, and its potential to deliver therapeutics. The idea of delivering antibiotics [29] or chemotherapeutics [30] locally in biomaterials has been presented since the early 1970s and late 1980s, respectively. While delivery of antibiotics has become standard care in some cases of orthopedic surgery [31], local delivery of chemotherapeutics has not been as widely applied clinically. We have previously demonstrated that local delivery of bisphosphonates is advantageous for disrupting bone metastases-induced osteolysis compared to systemic delivery [15], and furthermore we have shown that low-cost 3D printed scaffolds may be useful for orthopedic applications [19]. In this study, we established EC50 doses of doxorubicin for direct treatment of the prostate cancer cell line LAPC4 and patient derived spine metastases cells. Furthermore, we demonstrated that nanoporous 3D printed scaffolds can be used to effectively deliver doxorubicin to LAPC4 prostate cancer cells as well as patient derived spine metastases cells secondary to prostate cancer. The amount of doxorubicin released by the scaffolds could be modulated by the type of material used—the higher the porosity, the more drug could be released. Therefore, we have established a baseline for using the PORO-LAY 3D printed materials as a chemotherapeutic delivery device.

Common chemotherapeutics such as paclitaxel, docetaxel, cisplatin, daunorubicin, and doxorubicin are often administered systemically, and each one carries potentially severe side effects [32]. Many studies have therefore explored new ways to target therapeutics to the tumor sites to enhance drug effectiveness while reducing the negative side effects. One popular approach is targeted delivery of chemotherapeutics using nanoparticle carriers. Nanoparticles typically in the form of silica, lipids, iron oxide, polymers, or gold in the size range of 10 to 1000 nm [33], have been used to deliver drugs such as doxorubicin, cisplatin, and docetaxel. Bone targeting of doxorubicin loaded nanoparticles has been achieved by chemically linking bisphosphonates to the nanoparticles [34]. In other cases, therapeutics can be released near the tumor sites when temperature or pH changes occur [33]. While these methods potentially reduce the side effects of high systemic doses, they are still circulating systemically. Also, they do not solve the clinical problems associated with large tissue defects following tumor resections. 3D printing holds an advantage in that scaffolds can fill large defects, provide mechanical support, promote tissue repair and delivery antitumor therapeutics.

Since the expiration of several key patents in the last 5–10 years, low-cost 3D printers have become a highly common piece of equipment in industrial labs, academic labs and even in homes of laypersons and “tinkerers”. The devices are commonly used for rapid prototyping of conceptual ideas into testable models. Among the main types of additive manufacturing, fused deposition modeling is one of the least expensive platforms [35]. This type of 3D printing can use many different thermoplastic filaments such as the FDA approved materials PLA and PCL. We, and others, have shown the biocompatibility of 3D printed PLA [19,36,37,38,39] and PCL [40,41,42,43,44] scaffolds in vitro and in vivo. Based on its ease of access, rapid prototyping capabilities, use of FDA approved materials, mechanically competent structure design and potential for composite or chemically modified scaffold generation, the use of this technology will only grow in tissue engineering applications.

We focused our study on delivery of doxorubicin due to its wide clinical use and ease of in vitro monitoring by fluorescence. Several studies have shown that polymeric nanoparticles such as poly(lactic-co-glycolic acid) (PLGA), polyethylene glycol (PEG), and poly(caprolactone) (PCL) can be used for doxorubicin delivery [45]. Doxorubicin-loaded calcium phosphate cements were applied to bone tumor resection sites showing promising repair in bone sarcoma model [46], indicating that we are exploring a valid approach. We used porous 3D print filaments which have decreasing porosity from Gel Lay to Lay FOMM 40 to Lay FOMM 60, as evident in the presented SEM imaging. Our results show that choosing which PORO-LAY material can control doxorubicin release. Using a multihead 3D printer, composite scaffolds of the three materials could be designed to even further regulate the release of doxorubicin. Here, Gel Lay was most effective in treating a human prostate cancer cell line as well as patient derived bone metastases cells secondary to prostate cancer. Inexpensive 3D printing holds promise in generating patient-specific porous implants for precision treatment to fill bone defects while possibly limiting cancer recurrence. In vivo testing in bone tumor animal models using these scaffolds in combination with doxorubicin and other standard chemotherapeutics will be a crucial step in any clinical translation.

Sustained drug release is a critical component for maintaining high local doses of therapeutics using scaffold delivery systems. Our results indicate that approximately 60–75% of loaded doxorubicin could be released from our nanoporous scaffolds after 7 days in vitro without any modifications, indicating a potential for designing a sustained release system. Unmodified 3D printed PCL scaffolds have been shown to release ~95% of loaded doxorubicin within 4 days [47], which could then be modified with clay particles for sustained DOX release over 3 to 4 months. The same group showed that this 3D printed composite device was effective at treating tumors in vivo [48]. The drug release kinetics of the PORO-LAY scaffolds likely could be further controlled by coating, for example, with a chitosan shell as we have previously shown with silica nanoparticles [49,50]. Nonetheless, compared to the uncoated PLC 3D printed scaffolds [47], the nanoporous 3D printed scaffolds presented here hold the advantage of longer DOX release kinetics without the need for modifications.

While the scaffolds we have assessed in this study show strong promise for custom design and DOX uptake and release at effective rates for cancer cell lines and patient derived tumor cells, there are a few limitations that must be overcome in future work. The first limitation is that the PORO-LAY scaffolds are made from a proprietary blend of polyurethane and polyvinyl alcohol. While polyvinyl alcohol is well known to be food safe and nontoxic [51], compatibility and use of 3D printed polyurethane scaffolds is only beginning to gain attention [52,53,54]. Polyurethanes have long been used in biomedical tubing, catheters, and for some time, in breast and skin medical applications. The polymer is FDA approved and has been used in coating silicone breast implants for more than 25 years with no serious adverse effects observed in literature to date [55]. Medical grade polyurethane polymers have also been used safely in animal studies without any observed local or systemic side-effects [56]. While some formulations synthesized from aromatic diisocyanate may breakdown into carcinogenic components, many polyurethanes can be synthesized from resorbable, biocompatible polyesters such as FDA approved PCL, PLA, or PGA [57]. The polyurethanes used in our study are proprietary, and the information on their synthesis is not available to us. Nonetheless, our data indicate that they do not possess cytotoxic qualities on their own, after 7 days treatment to cells.

One limitation with the presented 3D printed scaffolds is their undefined mechanical properties. Although we did not quantitatively perform mechanical testing, it was blatantly obvious that neither of the three tested materials are rigid or possess mechanical stiffness comparable to bone. Therefore, in the current state, our scaffolds would be of little use as a bone substitute in a load-bearing defect site. However, using a composite scaffold approach, the PORO-LAY materials can be co-printed with PCL or PLA using layering and geometric designs conducive to optimal mechanical properties while retaining the ability to load and deliver therapeutics. This would be a similar approach to that of Chen et al., in their design of the 3D printed TPU/PLA/Graphene Oxide composite scaffold [54]. Furthermore, composite scaffolds can be designed whereby outer portions of the scaffold release more drug in a burst fashion (using Gel Lay) while inner portions release less drug over a longer, sustained period (using Lay FOMM 60). Another limitation in this study was assessment of sustained 0.01 µM DOX treatment of noncancerous cells. While local delivery of chemotherapeutics will presumably reduce side-effects associated with systemic delivery, it is important to understand whether prolonged low-dose DOX is cytotoxic to the nontumor cells in the immediate surrounding tissue. One study has indicated that DOX treatment using concentrations between 1 to 1.5 orders of magnitude higher that what we present here, do not inhibit normal cell metabolic activity in the same way as cancerous cells [58]. Future work will focus on increasing the mechanical strength and composition of the scaffolds and testing their efficacy in a xenograft animal model of bone metastases. Other future studies will also focus on co-delivery of chemotherapeutics with bioactive peptides or antiresorptive which may enhance bone repair while limiting cancer growth, as well as testing low-dose DOX on noncancerous primary human osteoblasts, stromal cells, and fibroblasts.

## Figures and Tables

**Table 1:** Comparison of metabolic activity assays of Dox-treated LAPC4 cells.

Cell Type	Alamar Blue			MTT		
	Ratio to Control *	SD	*p* Value	Ratio to Control *	SD	*p* Value
LAPC4	0.57	0.12	0.0025	0.96	0.14	0.29
Patient Cells	0.35	0.098	0.0002	0.91	0.13	0.14

* Ratio of assay activity with treatment of 0.01 µM Dox compared to control.
